# Caregiver burden and its related factors in advanced Parkinson’s disease: data from the PREDICT study

**DOI:** 10.1007/s00415-018-8816-9

**Published:** 2018-03-07

**Authors:** Alessandro Tessitore, Pietro Marano, Nicola Modugno, Francesco E. Pontieri, Nicola Tambasco, Margherita Canesi, Anna Latorre, Leonardo Lopiano, Mariachiara Sensi, Rocco Quatrale, Paolo Solla, Giovanni Defazio, Gabriella Melzi, Anna Maria Costanzo, Giuliana Gualberti, Umberto di Luzio Paparatti, Angelo Antonini

**Affiliations:** 1 First Division of Neurology, University of Campania, “Luigi Vanvitelli”, Napoli, Italy; 2 Nuova Casa di Cura D’Anna, Palermo, Italy; 3 Neurology Unit, IRCCS Neuromed, Pozzilli, IS Italy; 4 Department NESMOS, “Sapienza” University, Sant’Andrea Hospital, Rome, Italy; 5 Perugia General Hospital and University of Perugia, Perugia, Italy; 6 Centro Specialistico Ortopedico Traumatologico G. Pini-CTO Milano, Milan, Italy; 7 Sapienza University, Rome, Italy; 8 Department of Neuroscience, University of Torino, Azienda Ospedaliero-Universitaria Città della Salute e della Scienza di Torino, Turin, Italy; 9 Neurology Unit, Hospital Sant’Anna Ferrara, Ferrara, Italy; 10 Neurology Unit, Hospital dell’Angelo, Mestre, VE Italy; 11 Neurology Unit, Policlinico Universitario Monserrato, Cagliari, Italy; 12 AbbVie Srl, SR 148 Pontina, 04011 Campoverde, LT Italy; 13 Parkinson and Movement Disorders Unit, Department of Neuroscience, University of Padua, Padua, Italy

**Keywords:** Advanced Parkinson’s disease, Levodopa/carbidopa, Intestinal infusion, Caregiver burden, Quality of life

## Abstract

**Introduction:**

Caring for a person with Parkinson’s disease (PD) is associated with an increased risk of psychiatric morbidity and persistent distress. The objective of this study was to describe the burden and the related factors of caregivers of advanced PD (APD) patients either treated with continuous dopaminergic delivery systems or standard therapy.

**Methods:**

This cross-sectional, epidemiologic study conducted in 13 Italian sites enrolled PD patients treated with continuous dopaminergic delivering systems [either levodopa/carbidopa intestinal gel (LCIG) infusion or continuous subcutaneous apomorphine infusion (CSAI)] or continuation of standard of care (SOC) with a caregiver. Patient quality of life (QoL) and caregiver burden were assessed using the Parkinson’s Disease Questionnaire (PDQ-8) and Zarit Burden Inventory (ZBI), respectively.

**Results:**

126 patients (mean age 69.3 ± 8 years) and their caregivers (mean age 57.9 ± 12.9) were enrolled. Most caregivers were spouses. Fifty-three patients were treated with LCIG, 19 with CSAI, and 54 with SOC. Mean ZBI scores were 29.6 ± 14.4 for LCIG, 35.8 ± 20.2 for CSAI, and 31.4 ± 16.0 for SOC. Caregivers of LCIG, CSAI, and SOC patients showed no burden or mild/moderate burden in 74, 53, and 63% of the cases, respectively. Mean PDQ-8 scores were 11.25 ± 5.67, 11.26 ± 5.55, and 14.22 ± 6.51 in LCIG, CSAI, and SOC patients. Neurologists considered patients “very much or much improved” in 89, 58, and 13% of the LCIG, CSAI, and SOC groups using the Clinical Global Impression–Global Improvement Scale. Predictors significantly associated with caregiver burden were patients and caregivers’ judgment of QoL and caregivers’ need to change work.

**Conclusions:**

Caregiver burden showed a tendency to be lower when patients are treated with LCIG than with CSAI or SOC.

## Introduction

Parkinson’s disease (PD) is a neurodegenerative disorder characterized by troublesome motor and non-motor complications, with a progressive loss of autonomy in performing basic activities of daily living, which negatively impacts quality of life (QoL) [1]. Recent data showed that the main motor symptoms impacting the health-related QoL of patients with PD include dyskinesia, motor fluctuations, axial impairment, and freezing of gait [2]. Regarding non-motor symptoms (NMS), depression, dementia, psychosis, cognitive impairment, apathy, and sleep disorders were found to be the most common determinants in reducing QoL and increasing disability [3–5].

Due to the progressive disease course, there is an increasing utilization of economic and social resources. Indeed, patients often require caregiver assistance for daily activities, personal safety, medication compliance, and social involvement, with a consequent impact on caregiver burden and QoL [4–8]. Most PD patients are cared for in their own home and spouses are the main informal caregivers [6]. Caring for a partner or family member with a progressive neurologic disease has been recognized to negatively impact a caregiver’s physical, emotional, and psychosocial well-being, with an increased risk of psychiatric morbidity and persistent distress [9–11]. Moreover, caregiver burden increases as a patient’s disease progresses, with speech difficulties and cognitive deterioration associated with poorer caregiver QoL [11–13].

Due to the aging population and related increasing emergence of chronic neurologic diseases, caregiver burden has become a major issue. However, few studies have examined the identification of factors that affect the stress and QoL of caregivers in PD. Further, modifications in QoL are better perceived by the caregiver rather than the patient, due to the cognitive impairment that may arise as the disease progresses [14]. Moreover, the role of the caregiver becomes progressively important throughout the disease course, when motor and non-motor complications occur and disease management becomes more complex.

During the advanced PD (APD) stage, motor and NMS, including autonomic and psychological complaints and postural instability, require continuous adjustment of different medications, such as levodopa, dopamine agonists, catechol-*o*-methyltransferase inhibitors, and type B monoamine oxidase inhibitors [2, 15].

Even in the presence of motor fluctuations and disabling dyskinesia, many APD patients still continue to be treated with standard of care (SOC). A recent survey indicated that approximately 90% of APD patients were taking PD medication 5–9 times per day; 10% were taking medication > 10 times per day [16]. The same study showed that only 4% of APD patients were very satisfied with their conventional PD therapy [16]. Adequate management of intricate therapeutic schedules is essential to preserve satisfactory QoL in the advanced disease stage; however, this usually requires complex disease management, including frequent medication adjustments [2, 15]. Therapeutic options in APD include device-aided interventions, such as deep brain stimulation (DBS), continuous subcutaneous apomorphine infusion (CSAI), and levodopa/carbidopa continuous intestinal infusion gel (LCIG).

This study was designed to describe the burden and the related factors of caregivers of APD patients either treated with continuous dopaminergic delivery systems (CSAI or LCIG) or SOC.

## Patients and methods

### Study design

This observational study was conducted at 13 Movement Disorder (MD) centers in Italy, according to a cross-sectional design. Consecutive APD patients and their caregivers were recruited during routine follow-up visits planned at the MD centers.

### Patient selection

Inclusion criteria were the presence of a familial (non-professional) adult caregiver who had provided regular daily assistance (≥ 3 h per day) to the patient for ≥ 6 months; adult patients with APD in treatment with any of the following: (1) optimized infusion therapies (LCIG or CSAI) started ≥ 6 months before enrollment but ≤ 3 years or (2) continuing oral SOC who were offered but refused LCIG or CSAI or who had a ≥ 3-h OFF period per day or > 25% of daily time spent in OFF as assessed by United Parkinson’s Disease Rating Scale (UPDRS)-IV item 39.

Exclusion criteria included: patient history or presence of any severe condition that might interfere with caregiver burden assessments; patients with DBS; previous treatment with LCIG, CSAI, or DBS; mild to severe cognitive dysfunction/dementia (i.e., Mini-Mental State Examination score < 24 or per clinical judgment), and stage 5 Hoehn & Yahr (H&Y) in OFF in the past 12 months.

The present study was approved by the ethics committee of each local health authority. Each patient provided informed consent. The study was conducted according to the International Conference on Harmonisation Good Clinical Practices.

### Assessments

The primary endpoint of this study was to evaluate caregiver burden and its related factors among caregivers of APD patients using the Zarit Caregiver Burden Interview (ZBI) score. The ZBI assesses the impact of the disease on caregiver’s emotional, physical, social, and financial well-being [17] using a 22-item questionnaire with a 5-point scale, from 0 (never) to 4 (nearly always) [6]. The ZBI total score is categorized as follows: 0–20 (little or no burden), 21–40 (mild to moderate burden), 41–60 (moderate to severe burden), and 61–88 (severe burden). Scores were further aggregated into two categories: ZBI total score from 0 to 40 (little-to-moderate burden) and from 41 to 88 (moderate to severe burden).

### Parkinson’s disease features

Data collected from PD patients (CSAI/LCIG and SOC groups) included socio-demographic characteristics, comorbidities, drugs and drug regimens, disease staging (H&Y), disease onset, motor fluctuations, time spent in OFF, non-motor symptoms, levodopa equivalent daily dose (LEDD, calculated using the levodopa equivalent calculator, http://www.parkinsonsmeasurement.org), and reason for switching to infusion therapies or for remaining in SOC. Information about the source of first knowledge about advanced treatment options, patient’s working capacity, and working habits changes was also collected.

### Measures on caregiver and economic resources

Demographic characteristics of caregivers included relationship with patient, educational level and employment status were collected. Caregivers were asked to complete a qualitative survey to understand the burden due to familial assistance, working capacity, and working habits changes. Physicians provided paper questionnaires to caregivers in an envelope and instructed them to complete the questionnaire in the outpatient room.

### Measures of quality of life and treatment satisfaction

Patient satisfaction with the current treatment and caregivers’ judgement on their life perception and social adaptation were collected. Patient-reported quality of life measure includes the disease-specific eight-item Parkinson’s Disease Questionnaire (PDQ-8) [18]; clinicians completed the Clinical Global Impression–Improvement Scale (CGI-I) to assess the effectiveness of current treatment [19].

### Statistical analysis

The per-protocol population (PP) was defined as all-enrolled subjects without a main protocol violation. All statistical tables, figures, and analyses were produced using SAS^®^ for Windows release 9.4 (64-bit) or later (SAS Institute Inc., Cary, NC, USA). Patient and caregiver profile data were analyzed only in PP population by means of descriptive statistics. Whenever necessary, normality was assessed by means of the Shapiro–Wilk test and with graphical methods. In case of non-normality, appropriate transformation of data was applied or a nonparametric test/model was adopted. A two-sided *p* < 0.05 was considered statistically significant.

In all the analyses involving ZBI total score, only questionnaires with responses to ≥ 18 of 22 questions were used, according to copyright holder instructions [17]. For missing data (in case of only ≤ 4 missing questions), the average scores from valid responses were rounded to the nearest integer and used to complete any missing fields. To evaluate differences in the burden, stress, and QoL of caregivers of patients either treated with a continuous dopaminergic delivery system (LCIG or CSAI) or SOC, the ZBI total score in the two treatment groups (“standard treatment” vs “LCIG/CSAI”) was compared by means of an analysis of variance (ANOVA) for unbalanced data. The ANOVA (GLM—Generalized Linear Models) model was fitted to evaluate ZBI total score differences.

The ZBI total score was analyzed by means of Pearson/Spearman correlation coefficient in its correlation with: age of patient and of caregiver, PD duration, duration of motor fluctuation, daily time spent in OFF, in motor fluctuations, in dyskinesia, CGI-I rate, and PDQ-8 total score. The Pearson test was used in case of normal distribution of both the considered variables. The normality of variables distribution was assessed by means of Shapiro–Wilk test.

An ANOVA (GLM) model was fitted to evaluate ZBI total score differences and to compute a correlation ratio with regards to each of the following single factors: gender of patient and of caregiver, presence of PD-associated symptoms, caregiver’s duration of assistance, caregiver’s change in work, caregiver’s change in capability to perform family duties and leisure activities, need of professional assistance, H&Y stage, caregiver’s time spent per day for assistance, UPDRS-IV item 39, patient’s judgment on QoL, subject’s working capacity, number of outpatient visits (access to emergency department hospitalizations in the past 6 months due to PD), caregivers’ judgment on their QoL, and on their capability to perform family duties and leisure activities under the present treatment.

In the multiple regression model, only the variables significantly associated (*p* < 0.25) with the caregiver burden (ZBI total score) were considered.

### Determination of sample size

A sample size of 120 caregivers/subjects (unbalanced according to a 1:2 ratio, 40 on SOC and 80 on CSAI or LCIG) was calculated to estimate a statistically significant difference in the mean ZBI score equal or superior to 13 points, with the assumptions of 80% power, alpha 0.05, and a standard deviation (SD) of 25. At the time of the protocol writing, only a previous study showed a pre-post difference of more than 22 points with a standard deviation of approx. 13 points during a prospective assessment with LCIG [20]. No other data were available for the other populations under study.

To avoid major bias in the selection of the population, the sites were asked to consecutively enroll all subjects that met the inclusion criteria attending outpatient visits.

## Results

### Demographics and clinical characteristics

Enrollment was performed from September 2014 to September 2015. Of the 131 patients enrolled, 5 were excluded from the analysis due to protocol violations; thus, 126 patients were included in the PP population (53, 19, and 54 patients in the LCIG, CSAI, and SOC groups).

Demographic characteristics of patients and caregivers, including relationship with patient, caregiver’s educational level, patient’s and caregiver’s employment status, are summarized in Table 1. There were no significant differences in age and gender between groups.

**Table 1:** Demographic characteristics of the patients and caregivers

	LCIG (*n* = 53)	CSAI (*n* = 19)	SOC (*n* = 54)
Patients			
Demographics			
Age (years); mean ± SD (range)	70.26 ± 7.1 (53–84)	66.0 ± 6.6 (54–77)	69.57 ± 9.1 (42–88)
Female; *n* (%)	28 (53%)	9 (47%)	25 (46%)
Male; *n* (%)	25 (47%)	10 (53%)	29 (54%)
Employment *status*; *N* (%)			
Worker	0	3 (16%)	5 (9%)
Retired	45 (85%)	16 (84%)	48 (89%)
House keeper	7 (13%)	0	1 (2%)
Unemployed	1 (2%)	0	0
Caregivers			
Demographics			
Age (years); mean ± SD (range)	59.19 ± 13.2 (36–84)	60.26 ± 12.9 (29–78)	55.89 ± 12.6 (29–85)
Female; *n* (%)	30 (56%)	15 (79%)	43 (80%)
Male; *n* (%)	23 (43%)	4 (21%)	11 (20%)
Patient relationship; *N* (%)			
Spouse	33 (62%)	13 (68%)	30 (56%)
Son/daughter	16 (30%)	4 (21%)	20 (37%)
Brother/sister	1 (2%)	0	3 (5%)
Other relative	3 (6%)	2 (11%)	1 (2%)
Educational level; *N* (%)			
Elementary/middle school	29 (55%)	9 (48%)	17 (31%)
High school	20 (38%)	5 (26%)	28 (52%)
Academic degree	4 (7%)	5 (26%)	9 (17%)
Employment status; *N* (%)	*N* = 51	*N* = 19	*N* = 54
Worker	14 (27%)	5 (26%)	20 (37%)
Retired	22 (43%)	7 (37%)	12 (22%)
Student	0	1 (5%)	1 (2%)
House keeper	13 (26%)	6 (32%)	16 (30%)
Unemployed	2 (4%)	0	5 (9%)
Caregiver assistance duration	*N* = 52	*N* = 18	*N* = 53
Since 6–12 months	7 (14%)	0	3 (6%)
≥ 12 months	45 (86%)	18 (100%)	50 (94%)
Time spent during the day for the assistance	*N* = 53	*N* = 19	*N* = 52
Day and night (24 h)	26 (49%)	12 (63%)	29 (56%)
During daytime	8 (15%)	4 (21%)	9 (17%)
From 3 to 6 h per day	19 (36%)	3 (16%)	14 (27%)

*LCIG* levodopa/carbidopa intestinal gel, *CSAI* subcutaneous apomorphine infusion, *SOC* standard of care, *SD* standard deviation

Most caregivers were women (69.84% of the total cases; mean age 57.94 ± 12.9 years) taking care of spouses (60.32%), followed by sons/daughters (31.75%). The majority of caregivers (92%) were providing assistance to the patient for ≥ 12 months, with 53.17% of them caregiving 24 h a day (day and night; Table 1).

The clinical characteristics for each treatment group are reported in Table 2. The age at PD diagnosis was similar in the three groups, while disease duration was significantly longer in the LCIG group (16.38 ± 5.8 years) compared with the SOC group (12.83 ± 5.08 years; *p* = 0.0003). Similarly, the duration of motor fluctuations was significantly higher in the LCIG group (8.36 ± 4.8 years) than in the SOC group (difference vs LCIG group, − 3.14 years; *p* < 0.0001) and CSAI group (difference vs LCIG group, − 2.89 years; *p* = 0.0099), as reported in Table 2. In the LCIG group, the duration of daily motor fluctuations was significantly lower (50%) compared with the SOC group (*p* < 0.0001), as were OFF periods (75 and 40% less in the LCIG group than the SOC and CSAI groups; *p* < 0.0001; Table 2). Similarly, 81% of LCIG patients had < 25% of daily time spent in OFF as assessed by UPDRS-IV item 39, while 82% of the SOC patients showed more time spent in OFF (26% up to 75% of the day). In the CSAI group, 47% of the patients had < 25% of daily time spent in OFF (Table 2). The H&Y staging distribution was different in the three groups of patients. In fact, in the LCIG group, 32% of the patients were in H&Y stage 2.5 and 40% were in H&Y stage 3, while 19 and 65% of patients continuing SOC were in stages 2.5 and 3, respectively. Non-motor symptoms affected the majority of patients (99%), without significant differences among the three groups as reported in Table 2.

**Table 2:** PD clinical characteristics and PD-associated symptoms/comorbidities

	LCIG (*n* = 53)	CSAI (*n* = 19)	SOC (*n* = 54)	*p* value*,**
Age at PD diagnosis (years), mean ± SD, (range)	53.89 ± 9.1 (32–72)	52.42 ± 6.9 (40–62)	56.74 ± 9.4 (33–76)	0.524* 0.113**
PD duration (years), mean ± SD, (range)	16.38 ± 5.8 (7–33)	13.58 ± 4.1 (8–21)	12.83 ± 5.1 (4–35)	0.085* 0.0003**
Age at onset of motor fluctuations (years), mean ± SD, (range)	61.91 ± 8.8 (37–78)	60.53 ± 5.9 (52–72)	64.35 ± 9.4 (40–86)	0.529* 0.168**
Duration of motor fluctuations (years), mean ± SD, (range)	8.36 ± 4.8 (1–26)	5.47 ± 3.5 (2–14)	5.22 ± 4.1 (1–25)	0.0099* < 0.000**1
Duration of motor fluctuations (h/day), mean ± SD, (range)	2.69 ± 2.5 (0–11)	2.79 ± 1.5 (0–5)	5.42 ± 2.6 (1–14)	0.362* < 0.0001**
Duration of OFF periods (h/day), mean ± SD, (range)	1.38 ± 1.5 (0–6)	2.32 ± 1.7 (0–8)	5.47 ± 2.2 (3–12)	0.009* < 0.0001**
Duration of dyskinesia (h/day), mean ± SD, (range)	2.92 ± 3 (0–12)	2.79 ± 2 (0–8)	2.85 ± 3 (0–12)	0.5424* 0.7358**
UPDRS-IV Item 39, *N* (%)				
Not present	6 (11%)	1 (5%)	0	0.037* < 0.001**
1–25%	37 (70%)	8 (42%)	9 (17%)	
26–50%	9 (17%)	8 (42%)	36 (67%)	
51–75%	1 (2%)	2 (11%)	8 (15%)	
76–100%	0	0	1 (2%)	
Hoehn & Yahr in OFF, *N* (%)				0.121* 0.039**
1	1 (2%)	0	0	
1.5	2 (4%)	0	1 (2%)	
2	5 (9%)	2 (11%)	0	
2.5	17 (32%)	13 (68%)	10 (19%)	
3	21 (40%)	3 (16%)	35 (65%)	
4	7 (13%)	1 (5%)	8 (15%)	
PD-associated symptoms N (%)	98%	100%	100%	0.547* 0.311**
Falls	28 (54%)	11 (58%)	31 (57%)	0.704* 0.364**
Confusion	18 (35%)	9 (47%)	17 (31%)	0.300* 0.784**
Forgetfulness	17 (33%)	7 (37%)	18 (33%)	0.705* 0.890**
Bladder control problems	26 (50%)	10 (53%)	27 (50%)	0.789* 0.922**
Increased sweating	26 (50%)	12 (63%)	22 (41%)	0.291* 0.397**
Sexual disturbances	10 (19%)	10 (53%)	20 (37%)	0.005* 0.036**
Apathy	14 (27%)	9 (47%)	16 (30%)	0.093* 0.711**
Having felt sad or in a depressed mood for longer than 2 weeks	13 (25%)	5 (26%)	14 (26%)	0.877* 0.868**
Hallucinations	10 (19%)	2 (11%)	11 (20%)	0.403* 0.845**
Anxiety	22 (42%)	12 (63%)	32 (59%)	0.105* 0.066**
Cognitive function decline	19 (37%)	4 (21%)	16 (30%)	0.235* 0.493**
Sleep disturbances	29 (56%)	11 (58%)	35 (65%)	0.811* 0.287**
Impulse control disorders	4 (8%)	4 (21%)	8 (15%)	0.108* 0.234**
Fatigue	31 (60%)	15 (79%)	34 (63%)	0.111* 0.636**

*LCIG vs CSAI; **LCIG vs SOC; NA, not available

### Caregiver burden and associated factors

**Fig. 1:**
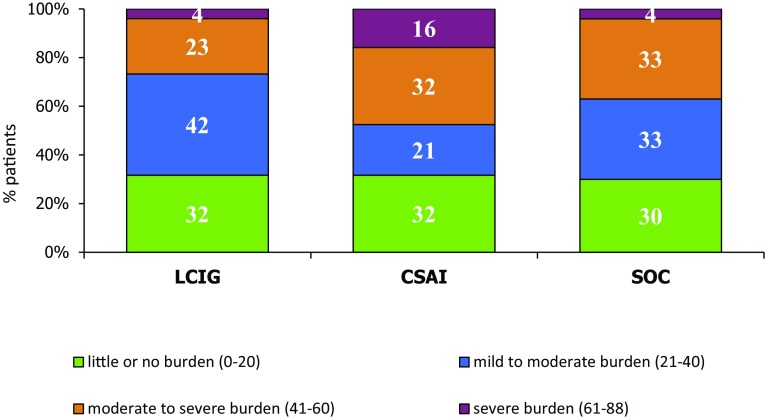
Distribution of ZBI-subscores in the three groups of caregivers

Mean ZBI scores did not differ significantly across groups, though lower scores were detected in the LCIG group (29.6 ± 14.42) compared with the CSAI (35.8 ± 20.15; *p* = 0.328 vs LCIG) and SOC groups (31.4 ± 16; *p* = 0.535 vs LCIG). The percentage of caregivers of LCIG patients showing no burden or little-to-moderate burden was higher (74%) than in the other groups (53 and 63% in the CSAI and SOC groups), but was not statistically significant (Fig. 1).

The Spearman correlation showed that the ZBI score was significantly correlated to the PDQ-8 score (*p* = 0.002), indicating that the patient’s life perception is related to caregiver burden. A statistically significant correlation was found between ZBI score and the following variables: “caregiver’s change in capability to perform family duties and leisure activities” (*p* < 0.001) and “caregiver’s change in work” (*p* = 0.001), “need of professional assistance” (*p* = 0.019), “patient’s judgment on QoL” (*p* = 0.010), and “caregiver’s judgment on QoL” (*p* < 0.001; Table 3).

**Table 3:** Correlation and association of ZBI score according to demographics and PD characteristics

ZBI total score and	*p* value
Spearman correlation	
Patient’s age (years)	0.906
Caregiver’s age (years)	0.935
PD duration (years)	0.811
Duration of motor fluctuations (years)	0.729
Duration of motor fluctuations (h/day)	0.736
Duration of dyskinesias (h/day)	0.470
Duration of OFF periods (h/day)	0.372
CGI-I score	0.303
PDQ-8 score	0.002
ANOVA models (univariate)	
Patient’s gender	0.380
Caregiver’s gender	0.093
Presence of PD-associated symptoms	0.924
Caregiver’s duration of assistance	0.347
Caregiver’s change in work	0.001
Caregiver’s change in capability to perform family duties/leisure activities	< 0.001
Need of professional assistance	0.019
Hoehn & Yahr stage	0.848
Caregiver’s time spent for assistance	0.168
UPDRS-IV item 39	0.474
Patient’s judgment on quality of life	0.010
Caregiver’s occupational status	0.577
No. of outpatient visits in the last 6 months due to PD	0.203
No. of accesses to emergency departments in the last 6 months due to PD	0.757
No. of hospitalizations in the last 6 months due to PD	0.415
Caregiver’s judgment on quality of life	< 0.001

Multiple regression linear analysis *(*model on ranks)	Estimate	*p* value	95% CI	
			Lower limit	Upper limit
Intercept	34.97	0.175	− 15.84	85.79
Caregiver’s change in work				
Change vs no change	23.57	0.001	10.38	36.76
Caregiver’s change in capability to perform family duties and leisure activities				
Change vs no change	9.21	0.072	− 0.83	19.25
Need of professional assistance				
Need vs no need	7.24	0.278	− 5.92	20.40
Patient’s judgment on quality of life				
Poor vs very poor	41.20	0.113	− 9.94	92.34
Good vs very poor	31.23	0.234	− 20.49	82.94
Very good vs very poor	21.88	0.416	− 31.28	75.04
Caregiver’s judgment on quality of life				
Poor vs very poor	− 3.48	0.737	− 24.04	17.08
Good vs very poor		0.003	− 53.28	
Very good vs very poor		0.005	− 59.01	

The multiple regression model showed a statistically significant association between the ZBI total score and the following variables: “caregiver’s change in work” (*p* = 0.001), “patient’s judgment on QoL,” (*p* = 0.0456, but it loses its statistical significance when ranks are compared), and “caregiver’s judgment on QoL” (*p* = 0.003 and 0.005 “good vs very poor” and “very good vs very poor”; Table 3).

### Patients’ treatment

The mean duration of continuous infusion at the time of the enrollment in the study was 17.8 ± 9.14 months in the LCIG group and 24.66 ± 9.94 in the CSAI group. The mean duration of LCIG infusion per day was 14.11 ± 2.08 h during daytime at a lower LEDD compared with that reported in the CSAI group (1112.64 ± 473.36 mg for LCIG and 1665.26 ± 1308.98 for CSAI; Table 4). As shown in Table 3, LCIG patients took a lower total number of anti-parkinsonian concomitant medication units per day (1.43 ± 0.65) than CSAI patients (6.69 ± 4.42; *p* = 0.001).

**Table 4:** Advanced therapeutic scheme in the two groups (LCIG and CSAI)

Parameter	LCIG (*N* = 53)	CSAI (*N* = 19)
Duration of infusion (h/day); mean ± SD (range)	14.1 ± 2.1 (11.0–24.0)	Not applicable
Levodopa equivalent daily dose (LEDD) (mg); mean ± SD (range)	1112.6 ± 473.4 (532–2960)	1665.3 ± 1309.0 (180–5600)
Duration of treatment (months); mean ± SD (range)	17.8 ± 9.1 (5.98–41.89)	24.7 ± 9.9 (5.65–40.15)
Patients using concomitant anti-parkinson medications during the day; *N* (%)	14 (26.4%)	13 (68.4%)
Number of anti-parkinson drug units/day; mean ± SD (range)	1.4 ± 0.7 (1–3)	6.7 ± 4.4 (1–19)

The majority of patients in the LCIG and CSAI groups used oral levodopa before switching to the advanced treatment (94 and 100%, respectively), with a LEDD of 972.5 ± 417.9 mg for the LCIG group and 1185 ± 686.3 for the CSAI group, while dopamine agonists were used in approximately 60% of both groups (Table 5). In the SOC group, the mean daily number of levodopa tablets per day was 5.59 ± 1.8 units, with a mean LEDD of 732.63 ± 230.1 mg.

**Table 5:** Standard treatment before LCIG or CSAI implementation and in patients continuing with SOC

Parameter	*N* (%)	No. of tablets/day (mean ± SD) (range)	LEDD (mg) (mean ± SD) (range)
Standard treatments before LCIG (*N* = 50)			
Oral levodopa	50 (94%)	6.0 ± 1.4 (2–9)	972.5 ± 417.9 (250–2350)
Dopamine agonists	33 (62%)	1.5 ± 0.7 (1–3)	274.6 ± 123.9 (100–560)
COMT inhibitors	23 (43%)	4.3 ± 1.9 (1–10)	322.6 ± 230.8 (100–1200)
MAO inhibitors	17 (32%)	1.1 ± 0.2 (1–2)	100.0 ± 0.0 (100–100)
Others^a^	12 (23%)	2.6 ± 2.4 (1–10)	204.3 ± 108.3 (100–360)
Standard treatments before CSAI (*N* = 19)			
Oral levodopa	19 (100%)	6.3 ± 1.8 (4–10)	1185 ± 686.3 (500–2500)
Dopamine agonists	12 (63%)	2.4 ± 2 (2–8)	437.2 ± 596.5 (105–2000)
COMT inhibitors	13 (68%)	4.4 ± 1.8 (2–8)	442.7 ± 403.1 (75–1400)
MAO inhibitors	3 (16%)	1 ± 0 (1–1)	100 ± 0 (100–100)
Amantadine	1 (5%)	2	200 (200–200)
Standard treatments in SOC-continuing patients (*N* = 54)			
Oral levodopa	54 (100%)	5.6 ± 1.8 (2–12)	732.6 ± 230.1 (200–1200)
Dopamine agonists	36 (67%)	1.4 ± 1.2 (1–8)	211.6 ± 81.0 (100–360)
COMT inhibitors	20 (37%)	4.5 ± 1.4 (2–7)	548.7 ± 414.0 (132–1400)
MAO inhibitors	19 (35%)	1.3 ± 1.2 (1–6)	237.5 ± 388.9 (100–1200)
Others^b^	15 (28%)	2.3 ± 1.3 (1–5)	293.2 ± 443.9 (100–1625)

*SD* standard deviation

^a^Others in LCIG group = amantadine (*n* = 9), apomorphine pen (*n* = 1), rasagiline (*n* = 1), rotigotine (*n* = 1)

^b^Others in the SOC group = amantadine (*n* = 11), levodopa/carbidopa/entacapone (*n* = 1), trihexyphenidyl hydrochloride (*n* = 1), clonazepam (*n* = 1)

### Attitude towards advanced therapies

Regarding the source of knowledge on the MD center managing the advanced treatment options reported by each treatment group, the main sources of information were community neurologists (26–40% of the cases) while patient associations as source of knowledge represented a small percentage of cases (4–11%; Table 6). Twenty-two percent of the caregivers assisting SOC patients declared that they had never received information about advanced therapies, while 43 and 35% were partially or constantly informed on this issue, respectively. Conversely, the majority of caregivers of LCIG or CSAI patients were fully informed about these treatments (Table 6).

**Table 6:** Source of knowledge for patients and caregiver on the advanced therapies and on MD implementing centers

	LCIG (*N* = 53)	CSAI (*N* = 19)	SOC (*N* = 54)
Information on MD centers			
General practitioner	11 (21%)	4 (21%)	13 (24%)
Community neurologist	21 (40%)	5 (26%)	15 (28%)
Media	7 (13%)	1 (5%)	5 (9%)
Patients’ association	2 (4%)	2 (11%)	4 (7%)
Others patients	13 (25%)	6 (32%)	12 (22%)
Other sources of information	11 (21%)	5 (26%)	12 (22%)

	LCIG (*N* = 53)	CSAI (*N* = 19)	SOC (*N* = 51)
Caregivers knowledge on advanced treatments			
Constantly informed	38 (72%)	17 (89%)	18 (35%)
Partially informed	13 (26%)	2 (11%)	22 (43%)
Not informed	2 (4%)	0	11 (22%)

Source of first information received on advanced PD treatment options		
Neurologist of the MD center	41 (77%)	16 (84%)
Community neurologist	7 (13%)	0
Media	1 (2%)	0
Other patients	2 (4%)	3 (16%)
General Practitioner	1 (2%)	0

Among patients receiving LCIG or CSAI, the main reasons that convinced them to switch from SOC to an infusion treatment were reduced QoL (79 and 84% in the LCIG and CSAI groups) or the presence of significant disability and loss of autonomy (85 and 68% in the LCIG and CSAI groups), as shown in Table 7. Conversely, the main reason why patients refused to initiate advanced therapies was fear to undergo invasive procedures (56% of the cases).

**Table 7:** Reasons for change from standard treatment to LCIG or CSAI and reasons to remain in SOC

Reasons for patients who decided to switch	LCIG (*N* = 53)	CSAI (*N* = 19)
	*N* (%)	*N* (%)
Caregiver was willing and available to assist patient in managing the advanced PD treatment for a long period	36 (68%)	14 (74%)
The patient could no longer tolerate poor quality of life	42 (79%)	16 (84%)
The patient presented significant disabilities, with serious loss of autonomy	45 (86%)	13 (68%)
The caregiver wished to reduce the stress he/she was undergoing	8 (15%)	3 (16%)
The patient and/or caregiver showed interest in advanced treatment options	23 (43%)	9 (47%)
Complexity of conventional treatments/scarce compliance	10 (19%)	3 (16%)

Reasons for patients to remain in standard treatment	SOC (*N* = 54)
	*N* (%)
The caregiver was not willing or available to assist patient in managing the advanced PD treatment for a long period	8 (15%)
The patient is afraid of advanced treatments	30 (56%)
The caregiver is worried for the patient	14 (26%)
The patient believes his or her conditions are not debilitating such as to require an advanced therapy	21 (39%)
The caregiver believes that advanced therapies have to be considered in the very late stage of disease	17 (31%)
The physician believes that the patient is unsuitable for an advanced therapy	17 (31%)
SOC patients who were proposed in the past an advanced therapy but they refused for any reason	18 (33%)

### Clinical judgment and quality of life perception

**Fig. 2:**
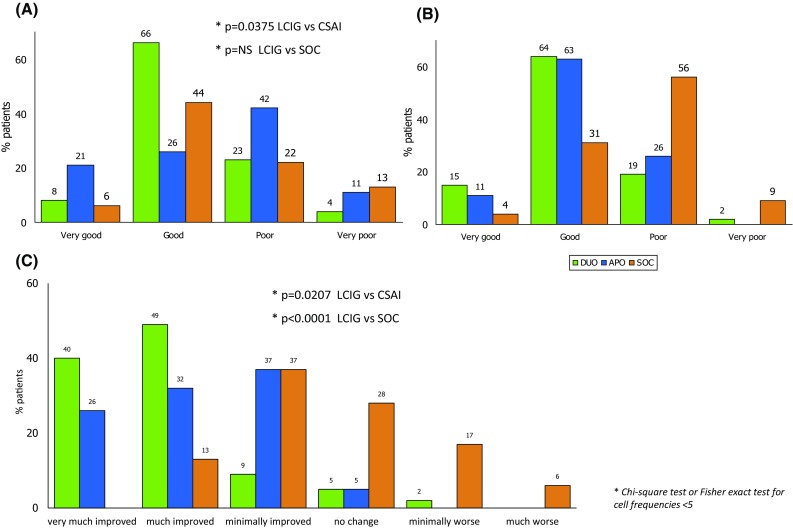
Global judgment of caregivers (**a**) and patients (**b**) on their current quality of life compared to previous standard treatment and Clinical Global Impression–Global Improvement Scale (CGI-I) by physician (**c**)

Within each treatment group, both patients and their caregivers expressed a similar judgment on their life perception. In fact, caregivers of LCIG patients rated their current life status as “good” or “very good” in the 74% of the cases, while such ratings were reported only by 47% of CSAI caregivers (*p* = 0.0375) and 50% of SOC caregivers (*p* = 0.117; Fig. 2a). Similarly, LCIG and CSAI patients rated their life as “good” or “very good” in 79 and 74% of the cases, respectively, with a lower percentage in the SOC group (35%). Moreover, 65% of the SOC patients described their life as “poor” or “very poor” (Fig. 2b). The clinicians considered 89% of the LCIG patients as “very much improved” or “improved,” with less improvement noted in the CSAI and SOC groups (58 and 13%, respectively; Fig. 2c). When patients treated with a continuous dopaminergic delivering system and their caregivers were asked to self-assess their current life compared with the previous SOC according to a score rated from 0 (very negative) to 10 (very positive), the mean score was 7.21 for LCIG patients and 6.37 for CSAI patients (LCIG vs CSAI; *p* = 0.02). In a similar way, the mean score expressed by the caregivers was 7.37 and 6.59, respectively, in LCIG and CSAI caregivers (LCIG vs CSAI, *p* = 0.0135).

**Fig. 3:**
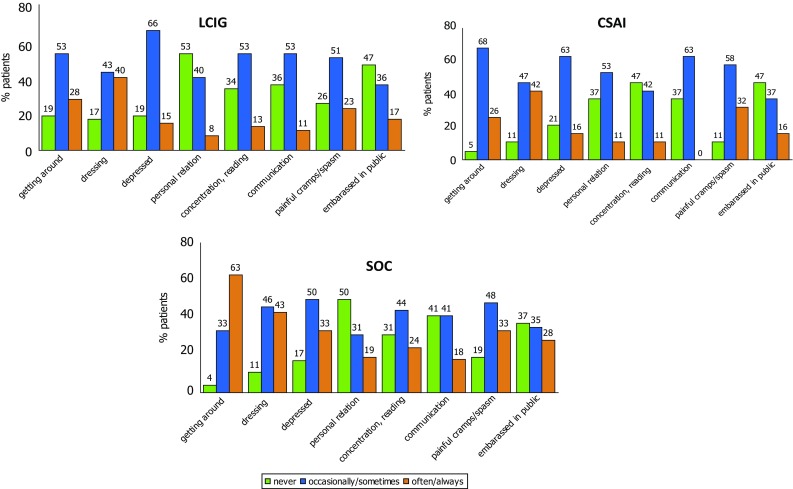
PDQ-8 subitems distribution in the three groups of patients

The mean PDQ-8 score was lower (indicating better QoL) in the LCIG and CSAI groups compared with the SOC group (11.25 ± 5.7 and 11.26 ± 5.6 vs 14.22 ± 6.5, respectively), with a statistically significant difference between LCIG and SOC (*p* = 0.013). Interestingly, as per the distribution of the single items of the PDQ-8 questionnaire reported in Fig. 3, LCIG patients had less difficulty getting around in public and felt less embarrassed in public compared with CSAI and SOC patients.

## Discussion

This observational study showed that caregiver burden is mainly associated to PD patient’s QoL, caregiver’s change in ability to perform family duties and leisure activities, caregiver’s change in work, the need of professional assistance, and by patient and caregiver judgment on life perception. The mean level of burden in each group is found to be in line with the ZBI range of 14.9–35.0 (SD = 9.9–18.7) reported in PD caregivers in previous studies [6]. We found a small reduction in caregiver burden in LCIG compared with CSAI and SOC caregivers. It is interesting to note that we found a mild level of caregiver burden (ZBI mean value = 29.6) in the LCIG group similar to that reported in recent studies (mean ZBI score varying from 21.8 to 28.3) where the caregivers had similar characteristics in terms of age, gender, familial relationship, and caregiving duration but with a shorter patients’ PD duration (8–9.7 years) compared to our study’s LCIG group [7, 8, 21]. Even though LCIG patients had a significantly higher disease duration (16.3 years) compared with CSAI or SOC patients (13.6 and 12.8 years, respectively) in our study, a higher level of caregiver burden was not documented. Moreover, these results take on more significance considering that the majority of caregivers were providing assistance for a longer period of time and half-invested 24 h per day in caregiving.

The PREDICT study also confirmed that a high percentage of patients in advanced stage disease (80% in H&Y stage 3 or 4) continues to be treated with SOC (> 5 oral levodopa units per day), even if most of them spent > 50% of daily time in the OFF state, with poor QoL in 65% of the patients in SOC and 41% of their caregivers, in addition to poor QoL reported by the treating physician. This finding is particularly relevant considering that the recently published Navigate-PD consensus reported that non-invasive therapies may be judged insufficiently when QoL becomes inadequate due to motor fluctuations with or without dyskinesias, and the clinician and patients agree that non-invasive therapy alone is no longer effective [22].

It is also interesting to note that, in our study, patients continuing with SOC were receiving > 5 oral levodopa intakes per day. This, together with the daily time spent in OFF, would be a reason to consider these patients as possible candidates for advanced therapies, as reported by the Navigate-PD consensus and Delphi panel [22, 23]. In fact, we also showed that patients switched to LCIG or CSAI were, on average, receiving six levodopa intakes per day. Both the consensus and the Delphi panel reported that patients requiring levodopa > 5 times daily, who have severe, troublesome OFF periods (> 1–2 h/day) despite optimal oral/transdermal levodopa or non–levodopa-based therapies, should be referred for specialist assessment, even if disease duration is < 4 years. It is also interesting to note that, in our study, some APD patients were receiving SOC and did not receive information about the possibility of disease management in MD centers. In fact, we found that this role is primarily devolved to community neurologists or MD specialists.

The main reason that patients receiving SOC did not switch to an advanced therapy was fear (56% of the cases); the main reasons for choosing advanced therapy were poor QoL, and the significant disability and loss of autonomy (approximately 80% of the patients).

It has been recently reported that NMS, sleep problems in particular, have a higher impact on caregiver burden and caregiver QoL than motor symptoms [24, 25]. Schrag et al. reported that 40% of caregivers experienced a deterioration in health due to NMS [11]. In our study, NMS, including sleep problems, fatigue, falls, urinary dysfunction, sweating, and anxiety, were frequently reported in each patient group. Notwithstanding the presence of NMS, 74% of LCIG caregivers expressed a good or very good perception of their own well-being, while in the CSAI and SOC groups, the percentages were lower. More caregivers of SOC patients were dissatisfied (41%) compared with those caring for LCIG patients (26%). The result obtained in LCIG caregivers is interesting, considering that > 50% of the caregivers of patients receiving DBS rated their subjective well-being as negative after 1 year of follow-up (especially if older and more depressed) [26]. In this study, patient QoL was significantly better in the LCIG and CSAI groups compared with the SOC group according to PDQ-8 scores. The improvement in QoL under LCIG over 24 months, using the PDQ-8, has been also recently reported in the GLORIA registry [27]. Moreover, both LCIG and CSAI patients had QoL that was good or very good, whereas 65% of SOC patients had poor or very poor QoL. These data are consistent with those reported by a Swedish survey on 3326 PD subjects, indicating that 67% of APD patients were unsatisfied with SOC [16].

Previous authors have reported that integrated clinical management of families with PD patients has become much more challenging from the physician’s perspective [26]. The development and introduction of new technologies and medications has been accompanied by strong involvement of patients and their families in overall care. Family members are more aware of the disease and treatment, and express their opinions and preferences at every step of the disease course. Therefore, the modern approach to PD management should include continuous engagement of patients and caregivers [13, 28]. This involves a redefinition of the patient–doctor relationship, creating a model of participatory medicine, with shared decision-making and helps provide care that is respectful of individual patient preferences and needs, including consensus on treatments to implement [29].

### Limitations and strengths

Our study extends knowledge on caregiver burden in APD. As reported in previous studies [6, 11], patient QoL is one of the main influencing factors of caregiver burden and the ZBI questionnaire should be considered as a valid instrument to assess the burden of PD. The main limitation of this study is its cross-sectional design, which did not allow consideration of the evolution of the burden of care and a comparison with baseline condition before treatment initiation, although data of the multiple regression analysis confirm the solidity of our findings. Moreover, the limited sample size in the CSAI group did not allow for a robust comparison with the LCIG group.

Another limitation of the study was the heterogeneous distribution of the H&Y stage in the three groups, which could have influenced both patients’ and caregivers’ answers. To limit this bias, we decided not to enroll patients in H&Y stage 5, because the answer given by the caregiver could be exclusively influenced by the disabling condition of the patient. Moreover, patients in H&Y stage 5 have a great difficulty in reaching the hospital to be routinely visited. Also the exclusion of patients with dementia or severe cognitive dysfunction could represent a further limitation.

## Conclusions

In summary, our study on caregiver burden offers interesting insights on the role of caregivers. Caregiver burden tends to be lower in familiars who assist patients treated with advanced therapies. Patient’s quality of life is frequently associated to caregiver burden which should be taken into account in the long-term management of PD.

Further research will help clarify whether the use of advanced therapies could reduce caregiver distress and the use of adjunctive socioeconomic resources.
