# Correction to: Caregiver burden and its related factors in advanced Parkinson’s disease: data from the PREDICT study

**DOI:** 10.1007/s00415-020-09833-0

**Published:** 2020-05-20

**Authors:** Alessandro Tessitore, Pietro Marano, Nicola Modugno, Francesco E. Pontieri, Nicola Tambasco, Margherita Canesi, Anna Latorre, Leonardo Lopiano, Mariachiara Sensi, Rocco Quatrale, Paolo Solla, Giovanni Defazio, Gabriella Melzi, Anna Maria Costanzo, Giuliana Gualberti, Umberto di Luzio Paparatti, Angelo Antonini

**Affiliations:** 1 First Division of Neurology, University of Campania, “Luigi Vanvitelli”, Napoli, Italy; 2 Nuova Casa di Cura D’Anna, Palermo, Italy; 3 Neurology Unit, IRCCS Neuromed, Pozzilli, IS Italy; 4 Department NESMOS, “Sapienza” University, Sant’Andrea Hospital, Rome, Italy; 5 Perugia General Hospital and University of Perugia, Perugia, Italy; 6 Centro Specialistico Ortopedico Traumatologico G. Pini-CTO Milano, Milan, Italy; 7 Sapienza University, Rome, Italy; 8 Department of Neuroscience, University of Torino, Azienda Ospedaliero-Universitaria Città della Salute e della Scienza di Torino, Turin, Italy; 9 Neurology Unit, Hospital Sant’Anna Ferrara, Ferrara, Italy; 10 Neurology Unit, Hospital dell’Angelo, Mestre, VE Italy; 11 Neurology Unit, Policlinico Universitario Monserrato, Cagliari, Italy; 12 AbbVie Srl, SR 148 Pontina, 04011 Campoverde, LT Italy; 13 Parkinson and Movement Disorders Unit, Department of Neuroscience, University of Padua, Padua, Italy

## Correction to: Journal of Neurology (2018) 265:1124–1137 https://doi.org/10.1007/s00415-018-8816-9

The original version of this article unfortunately contained a mistake. In Fig. 2, there are some mistakes that author would like to correct. In particular, in the part A of the figure we have added in the explanation of the figures that in the SOC group there were 8 subjects with missing data. In the part 2B we have included the statistical differences between groups while in the part 2C in the LCIG group, the subjects reporting no change in the CGI assessment was zero.

**Fig. 2:**
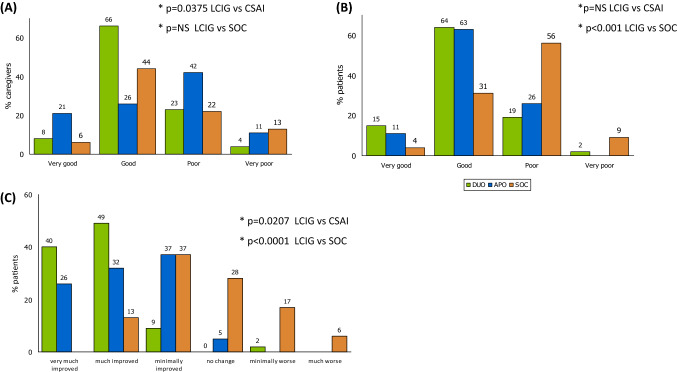
Global judgment of caregivers (**a**) and patients (**b**) on their current quality of life compared to previous standard treatment and Clinical Global Impression–Global Improvement Scale (CGI-I) by physician (**c**); * Chi-square test (**a**,** b**) or Fisher exact test (**c**); in **a**, **b** data were grouped as “very good + good” vs “poor + very poor”; in **c** data were grouped as “improved” vs “no change or worse”; 8 missing data for SOC caregivers in **a**

The corrected Fig. 2 with caption is placed.

